# At the Crossroads of Molecular Biology and Immunology: Molecular Pathways for Immunological Targeting of Head and Neck Squamous Cell Carcinoma

**DOI:** 10.3389/froh.2021.647980

**Published:** 2021-03-05

**Authors:** Niels E. Wondergem, Dennis N. L. M. Nijenhuis, Jos B. Poell, C. René Leemans, Ruud H. Brakenhoff, Rieneke van de Ven

**Affiliations:** ^1^Amsterdam UMC, Vrije Universiteit Amsterdam, Department of Otolaryngology/Head and Neck Surgery, Cancer Center Amsterdam, Amsterdam, Netherlands; ^2^Amsterdam Institute for Infection and Immunity, Amsterdam, Netherlands

**Keywords:** head and neck cancer, molecular targets, immune microenvironment, immunotherapy, signaling pathways

## Abstract

**Background:** Recent advances in immunotherapy for head and neck squamous cell carcinoma (HNSCC) have led to implementation of anti-programmed death receptor 1 (PD-1) immunotherapy to standard of care for recurrent/metastatic HNSCC. However, the majority of tumors do not respond to these therapies, indicating that these tumors are not immunogenic or other immunosuppressive mechanisms might be at play.

**Aim:** Given their role in carcinogenesis as well as in immune modulation, we discuss the relation between the STAT3, PI3K/AKT/mTOR and Wnt signaling pathways to identify potential targets to empower the immune response against HNSCC.

**Results:** We focused on three pathways. First, STAT3 is often overactivated in HNSCC and induces the secretion of immunosuppressive cytokines, thereby promoting recruitment of immune suppressive regulatory T cells and myeloid-derived suppressor cells to the tumor microenvironment (TME) while hampering the development of dendritic cells. Second, PI3K/AKT/mTOR mutational activation results in increased tumor proliferation but could also be important in HNSCC immune evasion due to the downregulation of components in the antigen-processing machinery. Third, canonical Wnt signaling is overactivated in >20% of HNSCC and could be an interesting pleotropic target since it is related to increased tumor cell proliferation and the development of an immunosuppressive HNSCC TME.

**Conclusion:** The molecular pathology of HNSCC is complex and heterogeneous, varying between sites and disease etiology (i.e., HPV). The in HNSCC widely affected signaling pathways STAT3, PI3K/AKT/mTOR and Wnt are implicated in some of the very mechanisms underlying immune evasion of HNSCC, thereby representing promising targets to possibly facilitate immunotherapy response.

## Introduction

In 2018, 700,000 patients were diagnosed with head and neck squamous cell carcinoma (HNSCC) worldwide, and 350,000 patients died of the disease [1]. HNSCC develops from squamous epithelial cells in the upper-aerodigestive tract, most frequently in the oral cavity, oropharynx, hypopharynx and larynx. The development of HNSCC is caused by alcohol and tobacco use [2]. Besides this, infection with human papillomavirus (HPV) is related to formation of a specific type of HNSCC tumors (HPV^+^) which are predominantly localized in the oropharynx and have distinct clinical, molecular and immunological characteristics when compared to HPV-unrelated (HPV^−^) tumors [3].

From a molecular point of view HNSCCs are very heterogeneous. Besides mutational changes in oncogenes and most particularly tumor suppressor genes, also epigenetic changes and chromosomal instability add to the overall molecular heterogeneity [3, 4]. This variable genetic background translates into a variety of tumor characteristics, challenges treatment efficacy and demands personalized approaches. Likewise, heterogeneity is observed in the immune composition of the tumor microenvironment (TME) of HNSCC, depending on etiology and/or localization of the tumor as recently reviewed by us and others [5–7]. In general, HPV^+^ HNSCC often display a more immune inflamed TME compared to HPV^−^ HNSCC, which are frequently immunologically cold or immune excluded. In addition, tumors with these separate etiologies seem to use different immune escape mechanisms [6, 7].

Despite advances in treatment, the survival rates for HNSCC have improved very moderately over the past five decades, with the average 5-year overall survival (OS) stabilizing at 40–60% for advanced stage disease [8]. Immunotherapy has been at the forefront of translational cancer research for the last decade and has provided great therapeutic benefits in the treatment of various cancer types [9], and also emerged as novel modality for HNSCC.

The currently available immunotherapies for treatment of HNSCC are nivolumab and pembrolizumab, which are both immune checkpoint inhibitors (ICIs) targeting the programmed death receptor 1 (PD-1). Nivolumab was approved in platinum refractory, recurrent/metastatic (R/M) HNSCC on basis of the results of the Checkmate-141 trial showing superior overall survival (OS) of the nivolumab treated arm when compared to investigator's choice of therapy (7.5 vs. 5.1 months; HR 0.70; 97.73% CI 0.51–0.96; *p* = 0.01) [10]. Pembrolizumab was first approved for platinum refractory R/M HNSCC based on the Keynote-012 trial that showed objective responses in 16% (95% CI 11–22) of patients, of which 5% were complete and 82% were durable (≥6 months) [11, 12]. The Keynote-048 trial has led to the approval of pembrolizumab as monotherapy or in combination with chemotherapy as first-line treatment for programmed death-ligand 1 (PD-L1) positive R/M HNSCC [13].

Many studies are underway investigating the use of these ICIs, and evaluating other immunotherapy regimens [14]. Recently promising results have been reported for pembrolizumab and nivolumab with or without ipilimumab [anti-cytotoxic T-lymphocyte–associated antigen 4 (CTLA-4)] neoadjuvant to surgery with curative intent in oral cavity squamous cell carcinoma [15, 16].

Currently, anti-PD-1 immunotherapy is effective in only a minority of HNSCC patients [17], and evidence accumulates that HNSCC can indeed be highly immune-evasive [18]. One approach is to study expression of immune-related genes in HNSCC that might relate to immune cell infiltration, predict patient outcome and could be applied to guide treatment choice [19]. Another approach to overcome immune-evasion might be to apply targeted therapies directed at molecular pathways known to be affected in HNSCC and that specifically relate to its immunological characteristics. To develop such targeted therapies, it is crucial to understand the specific pathways involved. In this review we relate the molecular landscape of HNSCC to its immunological characteristics, focusing on three of the most frequently affected pathways in HNSCC STAT3, PI3K/AKT/mTOR and Wnt, besides the EGFR pathway as a known target.

## Immunological Implications of the HNSCC Molecular Landscape

### EGFR

One of the major hallmarks of cancer is sustained proliferative signaling [20]. Growth factor signaling is commonly mediated by the family of receptor tyrosine kinase (RTK) cell-surface receptors, of which epidermal growth factor receptor (EGFR) is the most prominent in HNSCC [21]. It signals through the RAS-MAPK-, PI3K/AKT/mTOR-, phospholipase C-gamma-, signal transducers and activators of transcription (STAT)- and Src family kinase pathways [22]. Activation of EGFR promotes cell proliferation, angiogenesis, invasiveness and metastatic potential and has a prominent role in tumor initiation and maintenance. Activation of EGFR is seen in up to 80–90% of HNSCC cases as a result of *EGFR* amplifications or autocrine loops [23]. It should be noted, however, that *EGFR* does not show the typical activating mutations in HNSCC that are found in lung cancer, and whether HNSCC cells are really oncogene addicted remains elusive.

Cetuximab, a monoclonal antibody targeting EGFR, was the first new drug in decades, and the first targeted therapy, to be FDA approved for treatment of HNSCC [24]. Initially, cetuximab seemed quite promising with a favorable toxicity profile compared to chemotherapeutics, but recent phase III trials have demonstrated its inferiority to cisplatin in terms of primary disease control [25]. Cetuximab remains one of the cornerstones in the treatment of patients unfit to receive cisplatin and in the R/M setting [26].

While framed as a targeted drug and not introduced as an immunotherapy agent, cetuximab appeared to have an additional immune-related mode-of-action through the mediation of antibody-dependent cellular cytotoxicity (ADCC) [25]. Cetuximab stimulates the CD16/Fc receptor of natural killer (NK) cells resulting in their activation and the release of granzymes and perforins leading to tumor kill [25]. Increased interferon gamma (IFNγ) production by NK cells can induce PD-L1 expression on tumor cells and immune cells within the TME, providing a rationale for the possible synergistic potential of combining cetuximab with anti-PD-1 therapy [27–29]. Response to cetuximab treatment in HNSCC was shown to be hampered by treatment-induced recruitment of PD-1^+^ and TIM-3^+^ dysfunctional tumor-infiltrating T cells [30]. Also, PD-L1 expression on tumor cells hampered the cytolytic abilities of PD-1^+^ activated NK cells, reducing cetuximab efficacy in patients [31]. Preliminary data of a phase II trial evaluating the combination of PD-1 and EGFR inhibitory therapy showed promising results in 33 R/M HNSCC patients unfit for or refractory to cisplatin, with an overall response rate of 41% and few adverse events [32]. Results on other trials investigating the potential of this combinational regimen are to be awaited (Supplementary Table 1) [33].

### STAT3

**Figure 1:**
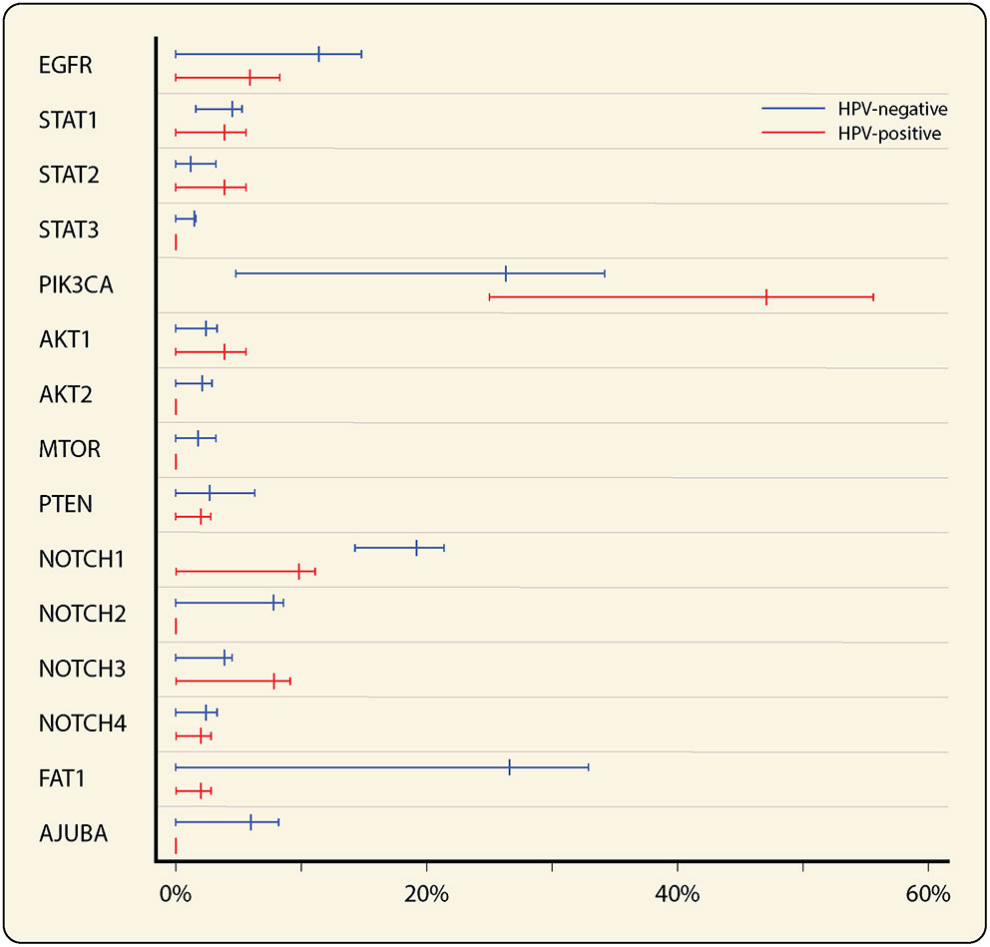
Forest plot of mutation rates of most frequent affected genes in EGFR, PI3K/AKT/mTOR and Wnt-signaling pathways in HNSCC, stratified by HPV status (total *n* = 385, 334 HPV^−^ and 51 HPV^+^). Mean mutation rates were estimated from the mutation rates in three HNSCC datasets [38–40], queried through cBioPortal [41, 42]. Minimum and maximum reported mutation rates are represented by the error bars.

STAT3 is part of the STAT protein family that regulates the transcription of various proliferative and cytokine-related genes [34]. It is of particular interest in HNSCC because of its near-universal signaling activation, already during early oral carcinogenesis [35], and its ability to be directly activated by EGFR [34]. As STAT3 mutations in HNSCC, leading to gain-of-function, have not been described, activation of STAT3 is presumably the result of enhanced signaling through its positive regulators (cytokines, growth factors and non-receptor TKs), or decreased signaling through its negative regulators (protein tyrosine phosphatase receptors) [36]. There are differences reported between HPV^+^ and HPV^−^ HNSCC with regards to STAT3 mutations and activation (Figure 1). Gaykalova et al. reported significantly more activated STAT3, as well as activated NF-κB, in HPV^−^ HNSCC [37].

**Figure 2:**
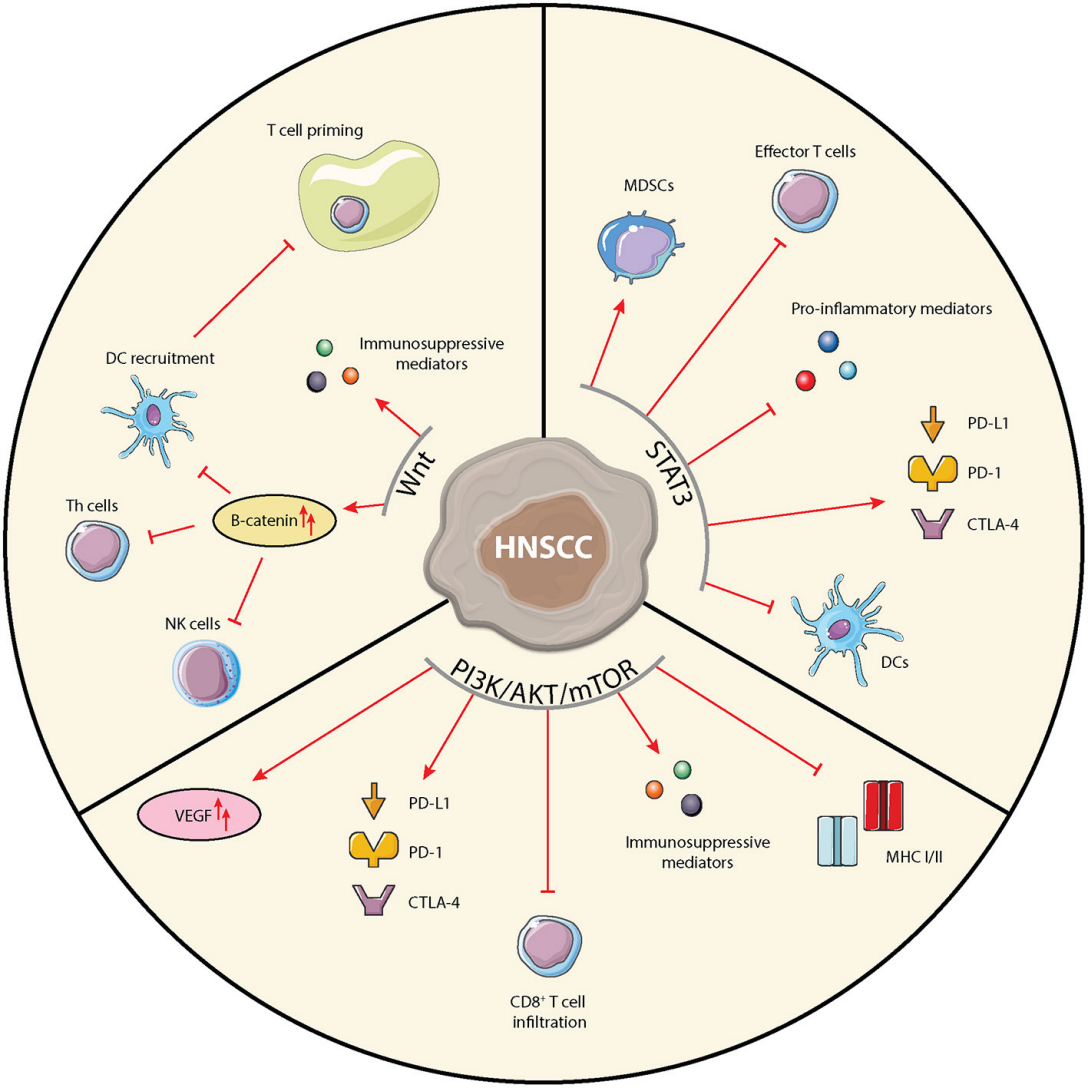
Schematic overview of immunological implications of the STAT3, PI3K/AKT/mTOR, and Wnt-signaling pathways in HNSCC cells. Arrows and bar-headed lines indicate stimulating and inhibiting effects, respectively, where red depicts pro-tumorigenic and green depicts anti-tumorigenic effects.

In addition to supporting tumor cell proliferation, STAT3 activity is related to a variety of immunosuppressive mechanisms and is a key regulator of immune processes (Figure 2) [43]. STAT3 inhibition could thus hit two birds with one stone. It is implicated in inhibition of pro-inflammatory mediators production such as IFNγ, antigen presentation, and accumulation and anti-tumor potential of effector T cells [44]. In myeloid-derived suppressor cells (MDSCs), STAT3 activation leads to expression of cyclin D and the S100A9 receptor, resulting in the suppression of cellular maturation and increased MDSC survival, respectively [45]. In the treatment of HNSCC, radiotherapy was shown to cause immune modulation by activating STAT3 in MDSCs [46]. Furthermore, STAT3 has the ability to increase expression of the PD-1/PD-L1/L2 and CTLA-4 checkpoints [44, 47].

Altogether, these data provide a rationale for combining STAT3 inhibitors with ICIs, which has already led to various pre-clinical and clinical studies [36, 44, 48]. AZD9150, a small DNA oligonucleotide which competitively blocks the binding site of STAT3 on its promotor has been tested in a phase I dose escalation study [49]. Results showed stable disease in 44% (*n* = 11/25) of all patients, and tumor shrinkage in 50% (*n* = 3/6) of treatment refractory lymphoma patients. In a follow-up study in treatment refractory lymphoma a clinical benefit was observed in 13% of patients [50]. A large study investigating AZD9150 combined with durvalumab anti-PD-L1 immunotherapy in patients with solid tumors including HNSCC is underway [51]. In a recent *in vitro* study in breast cancer sentinel lymph nodes (SLN), van Pul *et al*. showed that STAT3 inhibition in immune cells, combined with immune stimulation through TLR9 using CpG-B, could activate dendritic cell (DC) subsets in SLN cultures and increased tumor-specific T cell responses [52]. Using *in vivo* HNSCC mouse models, Moreira et al. showed that a STAT3-inhibiting oligonucleotide linked to CpG specifically targeted to myeloid cells, increased tumor sensitivity to radiotherapy and increased the anti-tumor immune response, suggesting that this could be a valid pathway to target in HNSCC [53]. In a syngeneic carcinogen-induced immune competent HNSCC mouse model, a small molecule inhibitor HNC0014, targeting cMET/STAT3/CD44 and PD-L1, was shown to reduce tumor growth, pSTAT3 and PD-L1 levels in tumors and increase T cell frequencies in the circulation most efficiently when combined with anti-PD-L1 treatment [48]. Of note, STAT3 inhibition was shown to reduce PD-L1 expression in HNSCC cell lines [47]. Potentially, STAT3 inhibition by itself might already reduce the inhibitory effect of the PD-1/PD-L1 axis, especially when tumor PD-L1 expression is regulated through oncogenic pathway activation often seen in HPV^−^ HNSCC. Combination therapy with ICIs targeting other immune checkpoints like TIM-3, LAG-3 or TIGIT might prove even more effective [54]. However, little is known at the moment about the effect of STAT3 on the expression of those immune checkpoints.

### PI3K/AKT/mTOR Pathway

The PI3K/AKT/mTOR pathway is involved in many cellular processes including cell cycle, survival, proliferation and motility [55]. Phosphatidylinositol 3-kinases (PI3Ks) are heterodimeric kinases formed by a regulatory and catalytic subunit and are activated by RTKs. The p110α catalytic subunit is encoded by a variety of genes of which *PIK3CA* is most important and harbors alterations in 26 and 47% of HPV^−^ and HPV^+^ HNSCC, respectively (Figure 1) [38–40]. Upon RTK activation, the regulatory subunit binds the catalytic subunit resulting in lipid phosphorylation and a cascade of events leading to activation of AKT, one of the major effectors of PI3K.

AKT is a serine-threonine kinase comprising three isoforms that are encoded by the *AKT1, AKT2*, and *AKT3* genes. Alterations in these genes are uncommon but overexpression of AKT in HNSCC has been reported as the result of a variety of factors such as microenvironmental stimuli, mutations in *PIK3CA* and diminished expression of PTEN [56–59]. PIK3CA and PTEN serve as central regulators of the PI3K/AKT/mTOR pathway and are known as bona fide HNSCC cancer genes.

The serine-threonine kinase mTOR comprises mTORC1 and mTORC2 and is an important downstream effector of AKT. It regulates critical biological functions including growth factor signaling and metabolism [55]. Genetic alterations in *EGFR, PIK3CA, PTEN*, and *HRAS* are known deregulators of mTOR signaling and are amongst the most frequently affected genes in HNSCC.

Various agents targeting PI3K, AKT, or mTOR have been studied in pre-clinical and clinical studies [60]. Although some studies in HNSCC have shown promising results, no trials have made it to phase III thus far due to unsatisfying efficacy and challenging tolerability. These early clinical trials have, however, provided valuable insights into the various physiological roles of the PI3K/AKT/mTOR pathway. It has become increasingly clear that apart from its functions in cancer cells, the PI3K/AKT/mTOR pathway also regulates many processes within the TME [61]. In various cancer types the PI3K/AKT/mTOR pathway has been implicated in the expression of immunosuppressive chemokines and cytokines [62, 63], expression of the immune suppressive vascular endothelial growth factor (VEGF) [64], reduced tumor CD8^+^ T cell infiltration [65], expression of immune checkpoints [66–68], and expression of MHC classes I and II (Figure 2) [69, 70]. In HNSCC the association of activated PI3K signaling with suppression of MHC expression was demonstrated using IHC, showing an inverse staining of MHC-I and phospho-AKT [71]. Moore et al. reported that co-inhibition of mTOR and PD-L1 enhanced anti-tumor efficacy in an oral cancer mouse model [72]. Since the PI3K/AKT/mTOR pathway is overactivated in over 90% of HNSCC, through various mechanisms, it is a highly interesting pathway to target in HNSCC, especially combined with ICIs [60].

### Wnt-Signaling

The Wnt-signaling cascade is an evolutionary highly conserved cascade important in (embryonic) cell growth, migration and differentiation [73]. About half of breast cancer tumors involve an activated Wnt-signaling pathway and hereditary colon cancer is often induced by a mutation in the adenomatous polyposis coli (*APC*) gene, part of the Wnt-signaling cascade [73]. Also in HNSCC, Wnt-signaling has been recognized as a central player [74].

Canonical Wnt-signaling depends on the APC destruction complex that regulates β-catenin levels, whereas non-canonical Wnt-signaling does not involve the APC complex. Binding of Wnt ligands to the Frizzled receptors leads to activation of the signaling pathway. When the Frizzled receptors are not stimulated by Wnt, cytoplasmic β-catenin levels are regulated by a destruction complex that includes amongst others APC, Axin and glycogen synthase kinase 3β (GSK3β) [73]. β-catenin is phosphorylated by GSK3β and subsequently degraded by the proteasome. Upon Frizzled receptor activation, however, Axin, APC and GSK3β are recruited to the membrane leading to inactivation of the destruction complex. Hence, β-catenin is able to translocate to the nucleus where it drives expression of target genes. These in turn regulate diverse cellular functions including cell proliferation, -survival and -migration [2].

β-catenin levels can be regulated by other processes as well, forming a source of non-canonical pathways. In HNSCC, several genes involved in Wnt-signaling are mutated or inactivated. The Cancer Genome Atlas (TCGA) data showed inactivating *FAT1* mutations in 23% of HNSCCs [38]. FAT1 is a cadherin-related adhesion receptor which can form cellular adhesion structures and therefore plays a role in cell-cell contact. Cadherin receptors can sequester β-catenin to the plasma membrane, thus preventing its translocation to the nucleus [75]. *FAT1* knockdown in glioblastoma cell lines resulted in promotion of cell cycle progression and cellular growth and in a breast cancer xenograft model *FAT1* knockdown resulted in the progression of ductal carcinoma *in situ* to invasive breast cancer [75, 76]. In HNSCC cell lines the effect of *FAT1* knockdown was inconsistent between studies, being associated with both increased [77] and decreased [78] migration and tumorigenesis. In HNSCC patients, *FAT1* mutations and downregulation are independent predictors of shorter disease-free survival [77]. Further studies are required to clarify the role of FAT1 in HNSCC carcinogenesis, including its effect on Wnt signaling.

AJUBA is a scaffold protein which stimulates the phosphorylation and degradation of β-catenin by activating GSK3β. Inactivating *AJUBA* mutations could result in a lower rate of β-catenin phosphorylation which in turn increases β-catenin-mediated gene transcription. Inactivating mutations in *AJUBA* are relatively infrequent in HNSCC with a prevalence of 6% and seem more common in HPV^−^ disease [38].

Also implicated in the Wnt-signaling pathway is Notch signaling. The Notch family comprises the Notch 1, Notch 2, Notch 3, and Notch 4 receptors, which are cleaved upon binding to their ligand through cell-cell contact, leading to the transfer of their intracellular domain into the nucleus to regulate the expression of target genes. The overall role of Notch signaling in cancer development is still highly debated. Mutational inactivation of Notch has been associated with tumor development, but overexpression has been associated with tumor progression and immune evasion [79]. *Notch 1* mutations are reported in 19% of HNSCC, most common in HPV^−^ tumors (Figure 1), and are correlated to worse outcome [80].

Loganathan et al. performed a CRISPR-Cas study in a HNSCC mouse model, in which they induced mutations in a wide-array of the so-called “long tail” genes [81], genes that are significantly mutated in tumors, but at very low frequency. Strikingly, mutational inactivation of p53 itself did not induce HNSCC, but introduction of mutations in these long tail genes did induce HNSCC tumor growth [81]. When evaluating the specific long tail genes it was found that knockout of *AJUBA* resulted in tumor growth but more importantly, it resulted in a significant decrease in Notch signaling [81]. Induction of Notch expression reversed the increased tumor growth of AJUBA-deficient mice, implicating Notch as the tumor-suppressive factor. Upon investigating the entire long tail gene panel, the authors ultimately concluded that in 67% of HNSCC cases, oncogenic mutations often impact Notch signaling, proposing decreased Notch signaling as a new hallmark of HNSCC [81].

The immunomodulatory role of the Wnt-signaling pathway in cancer has been reviewed extensively [82–85]. In line with its ubiquitous expression and involvement in many diverse cell functions, the Wnt pathway impacts many of the immunological players in the TME (Figure 2). Through activation of the Wnt-signaling pathway, DCs are pushed into a regulatory, tumor tolerant state. A recent study by Lopez-Gonzalez *et al*. showed that inhibition of Wnt-signaling through introduction of a constitutively active form of GSK3β restored human DC activation in the presence of immune suppressive cytokines and in an *in vivo* melanoma mouse model resulted in DC recruitment and activation and promoted tumor control [86]. Wnt-pathway activation in tumor cells negatively regulates the production of the chemokine CCL4, inhibiting the recruitment of cross-presenting DCs into the TME [87]. Consequently, infiltration of effector T cells into the TME is impaired, as no nodal T cell priming occurs. In melanoma cells, increased β-catenin expression was shown to even further hamper T cell priming through upregulation of IL-10 [88]. Indeed, analysis of TCGA data indicated that activation of the Wnt-pathway correlated with an immune-excluded TME in 90% of 31 included tumor types [89]. Additionally, upregulated β-catenin expression has been associated with impaired development and function of Th cells, impaired maturation of NK cells, and increased Treg survival [90]. Colombo et al. reviewed how cancer cells can use Notch signaling to change to cytokine milieu by secreting immune suppressive factors such as TGF-β, IL-10, CXCL12, thereby shaping a pro-tumorigenic microenvironment via cross-talk with stromal cells [91].

Hence, targeting Wnt-signaling seems a promising treatment strategy to augment ICI in HNSCC given its role in carcinogenesis as well as in shaping the TME [92]. Multiple small molecule inhibitors have been developed and some have already entered clinical trials also for HNSCC (Supplementary Table 1). LGK974 was shown to effectively inhibit *in vitro* HNSCC tumor growth and metastasis [93] and ICG001 was shown to downregulate HNSCC cancer stem cells and tumor growth *in vitro* [74]. Several other inhibitors of the Wnt-pathway are currently under clinical investigation [94].

Clinical trials targeting the Notch pathway have been performed and are currently ongoing [95]. However, inhibition of Notch is often associated with high toxicity possibly related to the notion that Notch signaling influences a wide array of processes [79, 81]. Clearly these issues need to be addressed, potentially by local rather than systemic inhibition of Notch, before Notch inhibitors could safely make their way to the clinic. To our knowledge, there are currently no targeted therapies available for *FAT1* or *AJUBA* mutated tumors. Obviously the notion that all these genes display inactivating mutations in HNSCC, hampers the exploitation as druggable target, and detailed functional characterization of the signaling pathways is crucial.

## Conclusion and Future Perspectives

We aimed to outline potential targets to aid HNSCC immunotherapy response by linking selected molecular pathways, widely-affected in HNSCC, to their immunological implications. STAT3 and the PI3K/AKT/mTOR- and Wnt-pathways are of particular interest, because they are implicated in some of the very mechanisms believed to be responsible for primary or acquired resistance to ICI therapy. Interfering with the immunomodulatory functions of these pathways could provide a means to boost the response to ICI therapies. Especially for HPV^−^ HNSCC, which is considered a immunologically “cold” tumor, such strategies may have promise. Cold tumors, characterized by an immune-excluded or -desert phenotype, are known to respond poorly to ICI [96], and could be pushed toward a more inflamed or “hot” phenotype by enabling CD8^+^ T cell infiltration through inhibition of the PI3K/AKT/mTOR or Wnt-pathways. Inhibiting these pathways would on the one hand inhibit the tumor cells, and on the other hand stimulate the immune cells. Moreover, the milieu of cytokines and other mediators including IFNγ and VEGF seems tailorable through the proposed pathways, as well as the expression of MHCI/II and immune checkpoints like PD-1/L1 and CTLA-4. The TME could additionally be organized into a more pro-inflammatory state through regulating the migration of immunosuppressive players, such as Tregs or MDSCs into the TME as well as directly mitigating their modulatory functions by targeting STAT3 both in the tumor cells and the immune cells. An overview of recent trials investigating agents targeting the discussed pathways is given in Supplementary Table 1. Given the difference in prevalence of alterations in particular molecular pathways between HPV^+^ and HPV^−^ HNSCC (Figure 1), certain targets might be more relevant for one of the etiologies. Interference with the STAT3 pathway could hold more clinical relevance for HPV^−^ disease, while in HPV^+^ disease the PI3K/AKT/mTOR pathway might be a more suitable target. Future clinical studies should consider building in a comparison between HPV^+^ and HPV^−^ HNSCC when evaluating the efficacy of drugs interfering with these molecular pathways. Moreover, it remains crucial to consider the fact that the functional consequences of targeting the discussed pathways could be unpredictable due to the complex composition of the HNSCC TME [97]. Dissecting the impact of targeted and combination therapies on the various cell populations involved in HNSCC may help explain the success or failure to potentiate an anti-tumor immune response.

## Author Contributions

NW and DN wrote the manuscript. JP, CL, RB, and RV guided and revised the manuscript. RV conceptualized and guided the project. All authors contributed to the article and approved the submitted version.

## Conflict of Interest

The authors declare that the research was conducted in the absence of any commercial or financial relationships that could be construed as a potential conflict of interest.

## References

[1] Bray F Ferlay J Soerjomataram I Siegel RL Torre LA Jemal A. Global cancer statistics 2018: GLOBOCAN estimates of incidence and mortality worldwide for 36 cancers in 185 countries. CA Cancer J Clin. (2018) 68:394–424. 10.3322/caac.21492 30207593

[2] Leemans CR Snijders PJF Brakenhoff RH. The molecular landscape of head and neck cancer. Nat Rev Cancer. (2018) 18:269–82. 10.1038/nrc.2018.11 29497144

[3] Leemans CR Braakhuis BJ Brakenhoff RH. The molecular biology of head and neck cancer. Nat Rev Cancer. (2011) 11:9–22. 10.1038/nrc2982 21160525

[4] Gaffey MJ Frierson HF Mills SE. Tumors of the Upper Aerodigestive Tract and Ear. 4th ed. Washington, DC: Armed Forces Institute of Pathology (2000).

[5] Bhat AA Yousuf P Wani NA Rizwan A Chauhan SS Siddiqi MA. Tumor microenvironment: an evil nexus promoting aggressive head and neck squamous cell carcinoma and avenue for targeted therapy. Signal Transduct Target Ther. (2021) 6:12. 10.1038/s41392-020-00419-w 33436555 PMC7804459

[6] Seliger B Massa C Yang B Bethmann D Kappler M Eckert AW. Immune escape mechanisms and their clinical relevance in head and neck squamous cell carcinoma. Int J Mol Sci. (2020) 21:7032. 10.3390/ijms21197032 32987799 PMC7582858

[7] Wondergem NE Nauta IH Muijlwijk T Leemans CR van de Ven R. The immune microenvironment in head and neck squamous cell carcinoma: on subsets and subsites. Curr Oncol Rep. (2020) 22:81. 10.1007/s11912-020-00938-3 32602047 PMC7324425

[8] Marur S Forastiere AA. Head and neck squamous cell carcinoma: update on epidemiology, diagnosis, and treatment. Mayo Clin Proc. (2016) 91:386–96. 10.1016/j.mayocp.2015.12.017 26944243

[9] Waldman AD Fritz JM Lenardo MJ. A guide to cancer immunotherapy: from T cell basic science to clinical practice. Nat Rev Immunol. (2020) 20:651–68. 10.1038/s41577-020-0306-5 32433532 PMC7238960

[10] Ferris RL Blumenschein G Jr. Fayette J Guigay J Colevas AD. Nivolumab for recurrent squamous-cell carcinoma of the head and neck. N Engl J Med. (2016) 375:1856–67. 10.1056/NEJMoa1602252 27718784 PMC5564292

[11] Seiwert TY Burtness B Mehra R Weiss J Berger R Eder JP. Safety and clinical activity of pembrolizumab for treatment of recurrent or metastatic squamous cell carcinoma of the head and neck (KEYNOTE-012): an open-label, multicentre, phase 1b trial. Lancet Oncol. (2016) 17:956–65. 10.1016/S1470-2045(16)30066-3 27247226

[12] Larkins E Blumenthal GM Yuan W He K Sridhara R Subramaniam S. FDA approval summary: pembrolizumab for the treatment of recurrent or metastatic head and neck squamous cell carcinoma with disease progression on or after platinum-containing chemotherapy. Oncologist. (2017) 22:873–8. 10.1634/theoncologist.2016-0496 28533473 PMC5507654

[13] Burtness B Harrington KJ Greil R Soulieres D Tahara M de Castro G. Pembrolizumab alone or with chemotherapy versus cetuximab with chemotherapy for recurrent or metastatic squamous cell carcinoma of the head and neck (KEYNOTE-048): a randomised, open-label, phase 3 study. Lancet. (2019) 394:1915–28. 10.1016/S0140-6736(19)32591-7 31679945

[14] Chow LQM. Head and neck cancer. N Engl J Med. (2020) 382:60–72. 10.1056/NEJMra1715715 31893516

[15] Uppaluri R Campbell KM Egloff AM Zolkind P Skidmore ZL Nussenbaum B. Neoadjuvant and adjuvant pembrolizumab in resectable locally advanced, human papillomavirus-unrelated head and neck cancer: a multicenter, phase II trial. Clin Cancer Res. (2020) 26:5140–52. 10.1158/1078-0432.CCR-20-1695 32665297 PMC7547532

[16] Schoenfeld JD Hanna GJ Jo VY Rawal B Chen YH Catalano PS. Neoadjuvant nivolumab or nivolumab plus ipilimumab in untreated oral cavity squamous cell carcinoma: a phase 2 open-label randomized clinical trial. JAMA Oncol. (2020). 10.1001/jamaoncol.2020.2955 PMC7453348 32852531

[17] Cramer JD Burtness B Le QT Ferris RL. The changing therapeutic landscape of head and neck cancer. Nat Rev Clin Oncol. (2019) 16:669–83. 10.1038/s41571-019-0227-z 31189965

[18] Kok VC. Current understanding of the mechanisms underlying immune evasion from PD-1/PD-L1 immune checkpoint blockade in head and neck cancer. Front Oncol. (2020) 10:268. 10.3389/fonc.2020.00268 32185135 PMC7058818

[19] Qiu Y Cui L Lin Y Gao B Li J Zhao X. Development and validation of a robust immune prognostic signature for head and neck squamous cell carcinoma. Front Oncol. (2020) 10:1502. 10.3389/fonc.2020.01502 33224866 PMC7667274

[20] Hanahan D Weinberg RA. Hallmarks of cancer: the next generation. Cell. (2011) 144:646–74. 10.1016/j.cell.2011.02.013 21376230

[21] Hynes NE Lane HA. ERBB receptors and cancer: the complexity of targeted inhibitors. Nat Rev Cancer. (2005) 5:341–54. 10.1038/nrc1609 15864276

[22] Yarden Y Pines G. The ERBB network: at last, cancer therapy meets systems biology. Nat Rev Cancer. (2012) 12:553–63. 10.1038/nrc3309 22785351

[23] Kalyankrishna S Grandis JR. Epidermal growth factor receptor biology in head and neck cancer. J Clin Oncol. (2006) 24:2666–72. 10.1200/JCO.2005.04.8306 16763281

[24] Bonner JA Harari PM Giralt J Azarnia N Shin DM Cohen RB. Radiotherapy plus cetuximab for squamous-cell carcinoma of the head and neck. N Engl J Med. (2006) 354:567–78. 10.1056/NEJMoa053422 16467544

[25] Moy JD Moskovitz JM Ferris RL. Biological mechanisms of immune escape and implications for immunotherapy in head and neck squamous cell carcinoma. Eur J Cancer. (2017) 76:152–66. 10.1016/j.ejca.2016.12.035 28324750 PMC5459368

[26] Vermorken JB Mesia R Rivera F Remenar E Kawecki A Rottey S. Platinum-based chemotherapy plus cetuximab in head and neck cancer. New Engl J Med. (2008) 359:1116–27. 10.1056/NEJMoa0802656 18784101

[27] Chen N Fang W Zhan J Hong S Tang Y Kang S. Upregulation of PD-L1 by EGFR activation mediates the immune escape in EGFR-driven NSCLC: implication for optional immune targeted therapy for NSCLC patients with EGFR mutation. J Thorac Oncol. (2015) 10:910–23. 10.1097/JTO.0000000000000500 25658629

[28] Azuma K Ota K Kawahara A Hattori S Iwama E Harada T. Association of PD-L1 overexpression with activating EGFR mutations in surgically resected nonsmall-cell lung cancer. Ann Oncol. (2014) 25:1935–40. 10.1093/annonc/mdu242 25009014

[29] Concha-Benavente F Srivastava RM Trivedi S Lei Y Chandran U Seethala RR. Identification of the cell-intrinsic and -extrinsic pathways downstream of EGFR and IFNgamma that induce PD-L1 expression in head and neck cancer. Cancer Res. (2016) 76:1031–43. 10.1158/0008-5472.CAN-15-2001 26676749 PMC4775348

[30] Jie HB Srivastava RM Argiris A Bauman JE Kane LP Ferris RL. Increased PD-1(+) and TIM-3(+) TILs during cetuximab therapy inversely correlate with response in head and neck cancer patients. Cancer Immunol Res. (2017) 5:408–16. 10.1158/2326-6066.CIR-16-0333 28408386 PMC5497750

[31] Concha-Benavente F Kansy B Moskovitz J Moy J Chandran U Ferris RL. PD-L1 mediates dysfunction in activated PD-1(+) NK cells in head and neck cancer patients. Cancer Immunol Res. (2018) 6:1548–60. 10.1158/2326-6066.CIR-18-0062 30282672 PMC6512340

[32] Sacco AG Chen R Ghosh D Wong DJ Worden FP Adkins D. An open label, nonrandomized, multi-arm, phase II trial evaluating pembrolizumab combined with cetuximab in patients with recurrent/metastatic (R/M) head and neck squamous cell carcinoma (HNSCC): Results of cohort 1 interim analysis. Am Soc Clin Oncol. 37:6033. (2019). 10.1200/JCO.2019.37.15_suppl.6033

[33] Saba NF Chen ZG Haigentz M Bossi P Rinaldo A Rodrigo JP. Targeting the EGFR and immune pathways in squamous cell carcinoma of the head and neck (SCCHN): forging a new alliance. Mol Cancer Ther. (2019) 18:1909–15. 10.1158/1535-7163.MCT-19-0214 31676542 PMC6830522

[34] Lo HW Hsu SC Ali-Seyed M Gunduz M Xia W Wei Y. Nuclear interaction of EGFR and STAT3 in the activation of the iNOS/NO pathway. Cancer Cell. (2005) 7:575–89. 10.1016/j.ccr.2005.05.007 15950906

[35] Grandis JR Drenning SD Zeng Q Watkins SC Melhem MF Endo S. Constitutive activation of Stat3 signaling abrogates apoptosis in squamous cell carcinogenesis *in vivo*. Proc Natl Acad Sci USA. (2000) 97:4227–32. 10.1073/pnas.97.8.4227 10760290 PMC18206

[36] Geiger JL Grandis JR Bauman JE. The STAT3 pathway as a therapeutic target in head and neck cancer: barriers and innovations. Oral Oncol. (2016) 56:84–92. 10.1016/j.oraloncology.2015.11.022 26733183 PMC5590227

[37] Gaykalova DA Manola JB Ozawa H Zizkova V Morton K Bishop JA. NF-kappaB and stat3 transcription factor signatures differentiate HPV-positive and HPV-negative head and neck squamous cell carcinoma. Int J Cancer. (2015) 137:1879–89. 10.1002/ijc.29558 25857630 PMC4629062

[38] Cancer Genome Atlas N. Comprehensive genomic characterization of head and neck squamous cell carcinomas. Nature. (2015) 517:576–82. 10.1038/nature14129 25631445 PMC4311405

[39] Agrawal N Frederick MJ Pickering CR Bettegowda C Chang K Li RJ. Exome sequencing of head and neck squamous cell carcinoma reveals inactivating mutations in NOTCH1. Science. (2011) 333:1154–7. 10.1126/science.1206923 21798897 PMC3162986

[40] Stransky N Egloff AM Tward AD Kostic AD Cibulskis K Sivachenko A. The mutational landscape of head and neck squamous cell carcinoma. Science. (2011) 333:1157–60. 10.1126/science.1208130 21798893 PMC3415217

[41] Cerami E Gao J Dogrusoz U Gross BE Sumer SO Aksoy BA. The cBio cancer genomics portal: an open platform for exploring multidimensional cancer genomics data. Cancer Discov. (2012) 2:401–4. 10.1158/2159-8290.CD-12-0095 22588877 PMC3956037

[42] Gao J Aksoy BA Dogrusoz U Dresdner G Gross B Sumer SO. Integrative analysis of complex cancer genomics and clinical profiles using the cBioPortal. Sci Signal. (2013) 6:pl1. 10.1126/scisignal.2004088 23550210 PMC4160307

[43] Rebe C Ghiringhelli F. STAT3, a master regulator of anti-tumor immune response. Cancers. (2019) 11:1280. 10.3390/cancers11091280 31480382 PMC6770459

[44] Zou S Tong Q Liu B Huang W Tian Y Fu X. Targeting STAT3 in cancer immunotherapy. Mol Cancer. (2020) 19:145. 10.1186/s12943-020-01258-7 32972405 PMC7513516

[45] Kumar V Patel S Tcyganov E Gabrilovich DI. The nature of myeloid-derived suppressor cells in the tumor microenvironment. Trends Immunol. (2016) 37:208–20. 10.1016/j.it.2016.01.004 26858199 PMC4775398

[46] Sampath S Won H Massarelli E Li M Frankel P Vora N. Combined modality radiation therapy promotes tolerogenic myeloid cell populations and STAT3-related gene expression in head and neck cancer patients. Oncotarget. (2018) 9:11279–90. 10.18632/oncotarget.24397 29541413 PMC5834279

[47] Bu LL Yu GT Wu L Mao L Deng WW Liu JF. STAT3 induces immunosuppression by upregulating PD-1/PD-L1 in HNSCC. J Dent Res. (2017) 96:1027–34. 10.1177/0022034517712435 28605599 PMC6728673

[48] Lee JC Wu ATH Chen JH Huang WY Lawal B Mokgautsi N. HNC0014, a multi-targeted small-molecule, inhibits head and neck squamous cell carcinoma by suppressing c-Met/STAT3/CD44/PD-L1 oncoimmune signature and eliciting antitumor immune responses. Cancers. (2020) 12:3759. 10.3390/cancers12123759 33327484 PMC7764918

[49] Hong D Kurzrock R Kim Y Woessner R Younes A Nemunaitis J. AZD9150, a next-generation antisense oligonucleotide inhibitor of STAT3 with early evidence of clinical activity in lymphoma and lung cancer. Sci Transl Med. (2015) 7:314ra185. 10.1126/scitranslmed.aac5272 26582900 PMC5279222

[50] Reilley MJ McCoon P Cook C Lyne P Kurzrock R Kim Y. STAT3 antisense oligonucleotide AZD9150 in a subset of patients with heavily pretreated lymphoma: results of a phase 1b trial. J Immunother Cancer. (2018) 6:119. 10.1186/s40425-018-0436-5 30446007 PMC6240242

[51] Cohen EEW Harrington KJ Hong DS Mesia R Brana I Perez Segura P. A phase Ib/II study (SCORES) of durvalumab (D) plus danvatirsen (DAN; AZD9150) or AZD5069 (CX2i) in advanced solid malignancies and recurrent/metastatic head and neck squamous cell carcinoma (RM-HNSCC): updated results. Ann Oncol. (2018) 29:viii372. 10.1093/annonc/mdy287

[52] van Pul KM Vuylsteke R de Beijer MTA van de Ven R van den Tol MP Stockmann H. Breast cancer-induced immune suppression in the sentinel lymph node is effectively countered by CpG-B in conjunction with inhibition of the JAK2/STAT3 pathway. J Immunother Cancer. (2020) 8:e000761. 10.1136/jitc-2020-000761 33046620 PMC7552844

[53] Moreira D Sampath S Won H White SV Su YL Alcantara M. Myeloid cell-targeted STAT3 inhibition sensitizes head and neck cancers to radiotherapy and T cell-mediated immunity. J Clin Invest. (2020) 131:e137001. 10.1172/JCI137001 33232304 PMC7810478

[54] Qin S Xu L Yi M Yu S Wu K Luo S. Novel immune checkpoint targets: moving beyond PD-1 and CTLA-4. Mol Cancer. (2019) 18:155. 10.1186/s12943-019-1091-2 31690319 PMC6833286

[55] Engelman JA. Targeting PI3K signalling in cancer: opportunities, challenges and limitations. Nat Rev Cancer. (2009) 9:550–62. 10.1038/nrc2664 19629070

[56] Amornphimoltham P Sriuranpong V Patel V Benavides F Conti CJ Sauk J. Persistent activation of the Akt pathway in head and neck squamous cell carcinoma: a potential target for UCN-01. Clin Cancer Res. (2004) 10(12 Pt 1):4029–37. 10.1158/1078-0432.CCR-03-0249 15217935

[57] Garcia-Carracedo D Villaronga MA Alvarez-Teijeiro S Hermida-Prado F Santamaria I Allonca E. Impact of PI3K/AKT/mTOR pathway activation on the prognosis of patients with head and neck squamous cell carcinomas. Oncotarget. (2016) 7:29780–93. 10.18632/oncotarget.8957 27119232 PMC5045433

[58] Bussink J van der Kogel AJ Kaanders JH. Activation of the PI3-K/AKT pathway and implications for radioresistance mechanisms in head and neck cancer. Lancet Oncol. (2008) 9:288–96. 10.1016/S1470-2045(08)70073-1 18308254

[59] Stegeman H Kaanders JH Wheeler DL van der Kogel AJ Verheijen MM Waaijer SJ. Activation of AKT by hypoxia: a potential target for hypoxic tumors of the head and neck. BMC Cancer. (2012) 12:463. 10.1186/1471-2407-12-463 23046567 PMC3517352

[60] Marquard FE Jucker M. PI3K/AKT/mTOR signaling as a molecular target in head and neck cancer. Biochem Pharmacol. (2020) 172:113729. 10.1016/j.bcp.2019.113729 31785230

[61] O'Donnell JS Massi D Teng MWL Mandala M. PI3K-AKT-mTOR inhibition in cancer immunotherapy, redux. Semin Cancer Biol. (2018) 48:91–103. 10.1016/j.semcancer.2017.04.015 28467889

[62] Ying H Elpek KG Vinjamoori A Zimmerman SM Chu GC Yan H. PTEN is a major tumor suppressor in pancreatic ductal adenocarcinoma and regulates an NF-kappaB-cytokine network. Cancer Discov. (2011) 1:158–69. 10.1158/2159-8290.CD-11-0031 21984975 PMC3186945

[63] Dong Y Richards JA Gupta R Aung PP Emley A Kluger Y. PTEN functions as a melanoma tumor suppressor by promoting host immune response. Oncogene. (2014) 33:4632–42. 10.1038/onc.2013.409 24141770

[64] Peng W Chen JQ Liu C Malu S Creasy C Tetzlaff MT. Loss of PTEN promotes resistance to T cell-mediated immunotherapy. Cancer Discov. (2016) 6:202–16. 10.1158/2159-8290.CD-15-0283 26645196 PMC4744499

[65] Bucheit AD Chen G Siroy A Tetzlaff M Broaddus R Milton D. Complete loss of PTEN protein expression correlates with shorter time to brain metastasis and survival in stage IIIB/C melanoma patients with BRAFV600 mutations. Clin Cancer Res. (2014) 20:5527–36. 10.1158/1078-0432.CCR-14-1027 25165098 PMC4216767

[66] Lastwika KJ Wilson W 3rd Li QK Norris J Xu H Ghazarian SR. Control of PD-L1 expression by oncogenic activation of the AKT-mTOR pathway in non-small cell lung cancer. Cancer Res. (2016) 76:227–38. 10.1158/0008-5472.CAN-14-3362 26637667

[67] Zhang C Duan Y Xia M Dong Y Chen Y Zheng L. TFEB mediates immune evasion and resistance to mTOR inhibition of renal cell carcinoma via induction of PD-L1. Clin Cancer Res. (2019) 25:6827–38. 10.1158/1078-0432.CCR-19-0733 31383732

[68] Deng L Qian G Zhang S Zheng H Fan S Lesinski GB. Inhibition of mTOR complex 1/p70 S6 kinase signaling elevates PD-L1 levels in human cancer cells through enhancing protein stabilization accompanied with enhanced beta-TrCP degradation. Oncogene. (2019) 38:6270–82. 10.1038/s41388-019-0877-4 31316145

[69] Marijt KA Sluijter M Blijleven L Tolmeijer SH Scheeren FA van der Burg SH. Metabolic stress in cancer cells induces immune escape through a PI3K-dependent blockade of IFNgamma receptor signaling. J Immunother Cancer. (2019) 7:152. 10.1186/s40425-019-0627-8 31196219 PMC6567539

[70] Sivaram N McLaughlin PA Han HV Petrenko O Jiang YP Ballou LM. Tumor-intrinsic PIK3CA represses tumor immunogenecity in a model of pancreatic cancer. J Clin Invest. (2019) 129:3264–76. 10.1172/JCI123540 31112530 PMC6668699

[71] Chandrasekaran S Sasaki M Scharer CD Kissick HT Patterson DG Magliocca KR. Phosphoinositide 3-Kinase signaling can modulate MHC Class I and II expression. Mol Cancer Res. (2019) 17:2395–409. 10.1158/1541-7786.MCR-19-0545 31548239 PMC7339488

[72] Moore EC Cash HA Caruso AM Uppaluri R Hodge JW Van Waes C. Enhanced tumor control with combination mTOR and PD-L1 inhibition in syngeneic oral cavity cancers. Cancer Immunol Res. (2016) 4:611–20. 10.1158/2326-6066.CIR-15-0252 27076449 PMC4930724

[73] Zhan T Rindtorff N Boutros M. Wnt signaling in cancer. Oncogene. (2017) 36:1461–73. 10.1038/onc.2016.304 27617575 PMC5357762

[74] Alamoud KA Kukuruzinska MA. Emerging insights into Wnt/beta-catenin signaling in head and neck cancer. J Dent Res. (2018) 97:665–73. 10.1177/0022034518771923 29771197 PMC11066518

[75] Morris LG Kaufman AM Gong Y Ramaswami D Walsh LA Turcan S. Recurrent somatic mutation of FAT1 in multiple human cancers leads to aberrant Wnt activation. Nat Genet. (2013) 45:253–61. 10.1038/ng.2538 23354438 PMC3729040

[76] Lee S Stewart S Nagtegaal I Luo J Wu Y Colditz G. Differentially expressed genes regulating the progression of ductal carcinoma in situ to invasive breast cancer. Cancer Res. (2012) 72:4574–86. 10.1158/0008-5472.CAN-12-0636 22751464 PMC3899801

[77] Lin SC Lin LH Yu SY Kao SY Chang KW Cheng HW. FAT1 somatic mutations in head and neck carcinoma are associated with tumor progression and survival. Carcinogenesis. (2018) 39:1320–30. 10.1093/carcin/bgy107 30102337

[78] Hsu TN Huang CM Huang CS Huang MS Yeh CT Chao TY. Targeting FAT1 inhibits carcinogenesis, induces oxidative stress and enhances cisplatin sensitivity through deregulation of LRP5/WNT2/GSS signaling axis in oral squamous cell carcinoma. Cancers. (2019) 11:1883. 10.3390/cancers11121883 31783581 PMC6966489

[79] Aster JC Pear WS Blacklow SC. The varied roles of notch in cancer. Annu Rev Pathol. (2017) 12:245–75. 10.1146/annurev-pathol-052016-100127 27959635 PMC5933931

[80] Zhao YY Yu GT Xiao T Hu J. The notch signaling pathway in head and neck squamous cell carcinoma: a meta-analysis. Adv Clin Exp Med. (2017) 26:881–7. 10.17219/acem/64000 29068587

[81] Loganathan SK Schleicher K Malik A Quevedo R Langille E Teng K. Rare driver mutations in head and neck squamous cell carcinomas converge on NOTCH signaling. Science. (2020) 367:1264–9. 10.1126/science.aax0902 32165588

[82] El-Sahli S Xie Y Wang L Liu S. Wnt signaling in cancer metabolism and immunity. Cancers. (2019) 11:904. 10.3390/cancers11070904 31261718 PMC6678221

[83] Wang B Tian T Kalland KH Ke X Qu Y. Targeting Wnt/beta-catenin signaling for cancer immunotherapy. Trends Pharmacol Sci. (2018) 39:648–58. 10.1016/j.tips.2018.03.008 29678298

[84] Goldsberry WN Londono A Randall TD Norian LA Arend RC. A review of the role of Wnt in cancer immunomodulation. Cancers. (2019) 11:771. 10.3390/cancers11060771 31167446 PMC6628296

[85] Martin-Orozco E Sanchez-Fernandez A Ortiz-Parra I Ayala-San Nicolas M. WNT signaling in tumors: the way to evade drugs and immunity. Front Immunol. (2019) 10:2854. 10.3389/fimmu.2019.02854 31921125 PMC6934036

[86] Lopez Gonzalez M Oosterhoff D Lindenberg JJ Milenova I Lougheed SM Martianez T. Constitutively active GSK3beta as a means to bolster dendritic cell functionality in the face of tumour-mediated immune suppression. Oncoimmunology. (2019) 8:e1631119. 10.1080/2162402X.2019.1631119 31646076 PMC6791458

[87] Spranger S Bao R Gajewski TF. Melanoma-intrinsic beta-catenin signalling prevents anti-tumour immunity. Nature. (2015) 523:231–5. 10.1038/nature14404 25970248

[88] Yaguchi T Goto Y Kido K Mochimaru H Sakurai T Tsukamoto N. Immune suppression and resistance mediated by constitutive activation of Wnt/beta-catenin signaling in human melanoma cells. J Immunol. (2012) 189:2110–7. 10.4049/jimmunol.1102282 22815287

[89] Luke JJ Bao R Sweis RF Spranger S Gajewski TF. WNT/beta-catenin pathway activation correlates with immune exclusion across human cancers. Clin Cancer Res. (2019) 25:3074–83. 10.1158/1078-0432.CCR-18-1942 30635339 PMC6522301

[90] Suryawanshi A Hussein MS Prasad PD Manicassamy S. Wnt signaling cascade in dendritic cells and regulation of anti-tumor immunity. Front Immunol. (2020) 11:122. 10.3389/fimmu.2020.00122 32132993 PMC7039855

[91] Colombo M Mirandola L Chiriva-Internati M Basile A Locati M Lesma E. Cancer cells exploit notch signaling to redefine a supportive cytokine milieu. Front Immunol. (2018) 9:1823. 10.3389/fimmu.2018.01823 30154786 PMC6102368

[92] Qian X Nie X Wollenberg B Sudhoff H Kaufmann AM Albers AE. Heterogeneity of head and neck squamous cell carcinoma stem cells. Adv Exp Med Biol. 11392019:23–40. 10.1007/978-3-030-14366-4_2 31134493

[93] Rudy SF Brenner JC Harris JL Liu J Che J Scott MV. *In vivo* Wnt pathway inhibition of human squamous cell carcinoma growth and metastasis in the chick chorioallantoic model. J Otolaryngol Head Neck Surg. (2016) 45:26. 10.1186/s40463-016-0140-8 27117272 PMC4845503

[94] Jung YS Park JI. Wnt signaling in cancer: therapeutic targeting of Wnt signaling beyond beta-catenin and the destruction complex. Exp Mol Med. (2020) 52:183–91. 10.1038/s12276-020-0380-6 32037398 PMC7062731

[95] Goruganthu MUL Shanker A Dikov MM Carbone DP. Specific targeting of notch ligand-receptor interactions to modulate immune responses: a review of clinical and preclinical findings. Front Immunol. (2020) 11:1958. 10.3389/fimmu.2020.01958 32922403 PMC7456812

[96] Wang HC Chan LP Cho SF. Targeting the immune microenvironment in the treatment of head and neck squamous cell carcinoma. Front Oncol. (2019) 9:1084. 10.3389/fonc.2019.01084 31681613 PMC6803444

[97] Peltanova B Raudenska M Masarik M. Effect of tumor microenvironment on pathogenesis of the head and neck squamous cell carcinoma: a systematic review. Mol Cancer. (2019) 18:63. 10.1186/s12943-019-0983-5 30927923 PMC6441173

