# Supplementary material for: At the Crossroads of Molecular Biology and Immunology: Molecular Pathways for Immunological Targeting of Head and Neck Squamous Cell Carcinoma
Source: Front Oral Health. 2021 Mar 5;2:647980. doi: 10.3389/froh.2021.647980 (PMC8757702; doi:10.3389/froh.2021.647980)
Supplement: Supplementary file 1 [file Table_1.DOCX]

| **Targeted pathway** | **Title** | **Drugs** | **Patients** | **Phase** | **NCT number** | **Status** | **Reference** |
| --- | --- | --- | --- | --- | --- | --- | --- |
| **EGFR** | Cetuximab & Nivolumab in Patients With Recurrent/Metastatic Head & Neck Squamous Cell Carcinoma | Cetuximab Nivolumab | HNSCC | I/II | NCT03370276 | Active, not recruiting | (1) |
|  | Nivolumab or Nivolumab Plus Cisplatin, in Combination With Radiotherapy in Patients With Cisplatin-ineligible or Eligible Locally Advanced Squamous Cell Head and Neck Cancer | Cetuximab Nivolumab | HNSCC | III | NCT03349710 | Completed | NA |
|  | Randomized Trial of Avelumab-cetuximab-radiotherapy Versus SOCs in LA SCCHN (REACH) | Cetuximab Avelumab | HNSCC | III | NCT02999087 | Active, not recruiting | (2) |
|  | EACH: Evaluating Avelumab in Combination with Cetuximab in Head and Neck Cancer | Cetuximab Avelumab | HNSCC | II | NCT03494322 | Active, not recruiting | (3) |
|  | Ipilimumab, Cetuximab, and Intensity-Modulated Radiation Therapy in Treating Patients With Previously Untreated Stage III-IVB Head and Neck Cancer | Cetuximab Ipilimumab | HNSCC | I | NCT01935921 | Active, not recruiting | (4) |
|  | Pembrolizumab Combined With Cetuximab for Treatment of Recurrent/Metastatic Head & Neck Squamous Cell Carcinoma | Cetuximab Pembrolizumab | HNSCC | II | NCT03082534 | Recruiting | (5) |
|  | Chemotherapy Plus Cetuximab in Combination With VTX-2337 in Patients With Recurrent or Metastatic Squamous Cell Carcinoma of the Head and Neck | VTX-2337 | HNSCC | II | NCT01836029 | Completed | (6) |
| **STAT3** | A Study of BBI608 in Adult Patients With Advanced Malignancies | BBI608 | Advanced malignancies | I | NCT01775423 | Completed | (7) |
|  | Oral STAT3 Inhibitor, TTI-101, in Patients With Advanced Cancers | TTI-101 | HNSCC Breast Cancer Non Small Cell Lung Cancer Hepatocellular Cancer Colorectal Cancer Gastric Adenocarcinoma Melanoma | I | NCT03195699 | Recruiting | NA |
|  | [STAT3 Inhibitor for Solid Tumors](https://clinicaltrials.gov/ct2/show/NCT00955812?term=NCT00955812&draw=2&rank=1) | OPB-31121 | Solid malignancies | I | NCT00955812 | Completed | (8) |
|  | Phase 1, Dose-escalation Trial of OPB-51602 in Patients With Advanced Solid Tumors | OPB-51602 | Solid malignancies | I | NCT01184807 | Completed | (9) |
|  | STAT3 DECOY in Head and Neck Cancer | STAT3 decoy | HNSCC | I | NCT00696176 | Completed | (10) |
|  | Study to Assess Safety, Tolerability and PK of AZD1480 in Patients With Solid Tumours | AZD-1480 | Solid malignancies | I | NCT01112397 | Terminated | NA |
|  | Study to Assess MEDI4736 With Either AZD9150 or AZD5069 in Advanced Solid Tumors & Relapsed Metastatic Squamous Cell Carcinoma of Head & Neck | AZD-9150 (danvatirsen)  AZD-5069 (CX2i)  MEDI4736 (durvalumab) | HNSCC Solid malignancies | I/II | NCT02499328 | Active, not recruiting | (11) |
| **PI3K/AKT/mTOR** | An Open Label, Single Arm, Multicenter Phase II Study of BYL719 in Patients With Recurrent or Metastatic Squamous Cell Carcinoma of Head and Neck Who Failed to Respond to Platinum-based Therapy | BYL719 (alpelisib) | HNSCC | ll | NCT02145312 | NA | NA |
|  | A Phase Ib/II Study of BYL719 and Cetuximab in Recurrent or Metastatic Head and Neck Squamous Cell Carcinoma | Cetuximab BYL719 (alpelisib) | HNSCC | l/ll | NCT01602315 | Terminated | NA |
|  | A Phase Ib, open-label, dose-finding study of alpelisib in combination with paclitaxel in patients with advanced solid tumors | Paclitaxel BYL719 (alpelisib) | HNSCC Breast cancer | l | NCT02051751 | Completed | (12) |
|  | BKM120 for Patients With PI3K-activated Tumors | BKM-120 (buparlisib) | HNSCC | l | NCT01833169 | Completed | (13) |
|  | Activity and Safety Study of BKM120 in Monotherapy in Patient With Metastatic Head and Neck Cancer Recurrent or Progressive (PIK-ORL) | BKM-120 (buparlisib) | HNSCC | ll | NCT01737450 | Completed | (14) |
|  | PI3K Inhibitor BKM120 and Cetuximab in Treating Patients With Recurrent or Metastatic Head and Neck Cancer | Cetuximab BKM-120 (buparlisib) | HNSCC | l/ll | NCT01816984 | Active, not recruiting | (15) |
|  | Study of Efficacy and Safety of Buparlisib (BKM120) Plus Paclitaxel Versus Placebo Plus Paclitaxel in Recurrent or Metastatic Head and Neck Cancer Previously Pre-treated With a Platinum Therapy | Paclitaxel BKM-120 (buparlisib) | HNSCC | ll | NCT01852292 | Terminated | (16) |
|  | A multicenter phase 1 study of PX-866 and cetuximab in patients with metastatic colorectal carcinoma or recurrent/metastatic squamous cell carcinoma of the head and neck | Cetuximab PX-866 | Colorectal carcinoma HNSCC | l | NCT01252628 | Completed | (17) |
|  | A randomized, phase 2 trial of docetaxel with or without PX-866, an irreversible oral phosphatidylinositol 3-kinase inhibitor, in patients with relapsed or metastatic head and neck squamous cell cancer | Docetaxel PX-866 | HNSCC | ll | NA |  | (18) |
|  | Copanlisib in Association With Cetuximab in Patients With Recurrent and/or Metastatic Head and Neck Squamous Cell Carcinomas Harboring a PI3KCA Mutation/Amplification and/or a PTEN Loss (COPAN-ORL06) | Cetuximab Copanlisib | HNSCC | l/ll | NCT02822482 | Active, not recruiting | NA |
|  | Study of the CDK4/6 Inhibitor Palbociclib (PD-0332991) in Combination With the PI3K/mTOR Inhibitor Gedatolisib (PF-05212384) for Patients With Advanced Squamous Cell Lung, Pancreatic, Head & Neck and Other Solid Tumors | PD-0332991 (palbociclib) PF-05212384 (gedatolisib) | Lung Cancer Squamous Cell HNSCC Pancreatic Cancer Solid Tumors | I | NCT03065062 | Recruiting | NA |
|  | SF1126 in Recurrent or Progressive SCCHN and Mutations in PIK3CA Gene and/or PI-3 Kinase Pathway Genes | SF1126 | HNSCC | ll | NCT02644122 | Terminated | NA |
| **Wnt** | A Phase II Trial Evaluating WNT974 in Patients With Metastatic Head and Neck Squamous Cell Carcinoma | WNT974 | HNSCC | II | NCT02649530 | Withdrawn | NA |
|  | A Study to Evaluate the Safety and Tolerability of ETC-1922159 in Advanced Solid Tumours | ETC-1922159 Pembrolizumab | Solid tumors | I | NCT02521844 | Recruiting | (19) |
|  | Study to Evaluate the Safety and Tolerability of RXC004 in Advanced Malignancies | RXC004 | Solid tumors | I | NCT03447470 | Recruiting | NA |
|  | CGX1321 in Subjects With Advanced Solid Tumors and CGX1321 With Pembrolizumab in Subjects With Advanced GI Tumors (Keynote 596) | CGX1321 Pembrolizumab | Solid tumors | I | NCT02675946 | Recruiting | NA |
|  | A Dose Escalation Study of OMP-18R5 in Subjects With Solid Tumors | OMP-18R5 (vantictumab) | Solid tumors | I | NCT01345201 | Completed | (20) |
|  | A Dose Escalation Study of OMP-54F28 in Subjects With Solid Tumors | OMP-54F28 (ipafricept) | Solid tumors | I | NCT01608867 | Completed | (21) |
|  | A Study Evaluating the Safety and Pharmacokinetics of Orally Administered SM08502 in Subjects With Advanced Solid Tumors | SM08502 | Solid tumors | I | NCT03355066 | Recruiting | NA |
| **Notch** | A Multiple Ascending Dose Study of R4733 in Patients with Advanced Solid Tumours | R4733 (RO4929097) | Solid tumors | I | NCT00532090 | Completed | NA |
|  | Gamma-Secretase Inhibitor RO4929097 in Treating Patients with Metastatic or Unresectable Solid Malignancies | R4733 (RO4929097) | Solid tumors | I | NCT01096355 | Completed | NA |
|  | Gamma-Secretase Inhibitor RO4929097 and Gemcitabine Hydrochloride in Treating Patients with Advanced Solid Tumours | R4733 (RO4929097) | Solid tumors | I | NCT01145456 | Completed | (22) |
|  | Gamma-Secretase/Notch Signalling Pathway Inhibitor RO4929097 and Temsirolimus in Treating Patients with Advanced Solid Tumours | R4733 (RO4929097) | Solid tumors | I | NCT01198184 | Completed | (23) |
|  | A Study of LY3039478 in Participants with Advanced Cancer | LY3039478 (JSMD194) | Solid tumors | I | NCT01695005 | Completed | (24) |
|  | A Phase 1b Study of LY3039478 in Combination with Other Anticancer Agents in Patients With Advanced or Metastatic Solid Tumours | LY3039478 (JSMD194) | Solid tumors | I | NCT02784795 | Completed | NA |
|  | A Phase I trial of PF-03084014 in patients with advanced solid tumour malignancy and T-cell acute lymphoblastic leukemia/lymphoblastic lymphoma | PF-03084014 (nirogacestat) | Solid tumors T cell acute lymphoblastic leukemia Lymphoblastic lymphoma | I | NCT00878189 | Completed | (25) |
|  | Study to Evaluate the Safety and Tolerability of IV Doses of BMS-906024 in Subjects with Advanced or Metastatic Solid Tumours | BMS-906024 | Solid tumors | I | NCT01292655 | Completed | (26) |
|  | Study to Evaluate Safety & Tolerability of BMS-906024 in Combination With Chemotherapy & to Define DLTs & MTD of BMS-906024 in Combination With One of the Following Chemotherapy Regimens; Weekly Paclitaxel, 5FU+Irinotecan or Carboplatin+Paclitaxel in Subjects With Advanced / Metastatic Solid Tumours | BMS-906024 | Solid tumors | I | NCT01653470 | Completed | NA |
|  | Phase I Ascending Multiple-Dose Study of BMS-986115 in Subjects with Advanced Solid Tumours | BMS-986115 | Solid tumors | I | NCT01986218 | Terminated | (27) |
|  | A Phase 1 Study to Evaluate the Safety, Tolerability, and Pharmacokinetics of MEDI0639 in Advanced Solid Tumours | MEDI0639 | Solid tumors | I | NCT01577745 | Completed | (28) |
|  | A Multiple-Ascending-Dose Study of the Safety and Tolerability of REGN421 (SAR153192) in Patients with Advanced Solid Malignancies | REGN421 (SAR153192) | Solid tumors | I | NCT00871559 | Completed | (29) |
|  | A Phase 1b Study of Demcizumab Plus Pembrolizumab in Locally Advanced or Metastatic Solid Tumours | Demcizumab | Solid tumors | I | NCT02722954 | Completed | (30) |
|  | A Dose Escalation Study of OMP-52M51 in Subjects with Solid Tumours | OMP-52M5 (brontictuzumab) | Solid tumors | I | NCT01778439 | Completed | (31) |
|  | A Dose Escalation Study of OMP-59R5 in Subjects with Solid Tumours | OMP-59R5 (tarextumab) | Solid tumors | I | NCT01277146 | Completed | (32) |
|  | A Dose Escalation Study of PF-06650808 in Patients With Advanced Solid Tumors | PF-06650808 | Solid tumors | I | NCT02129205 | Terminated | (33) |
|  | A Phase 1 Study of OMP-305B83 in Subjects With Solid Tumors | OMP-305B83 | Solid tumors | I | NCT02298387 | Completed | (34) |

**Supplementary Table 1.** Overview of recent (ongoing) clinical trials investigating agents targeting the EGFR, STAT3, PI3K/AKT/mTOR, Wnt and Notch signalling pathways. HNSCC: head and neck squamous cell carcinoma; NA: not available.

# References

1. Chung CH, Bonomi MR, Steuer CE, Schell MJ, Li J, Johnson M, et al. Concurrent cetuximab (CTX) and nivolumab (NIVO) in patients with recurrent and/or metastatic (R/M) head and neck squamous cell carcinoma (HNSCC): Results of phase II study. Journal of Clinical Oncology. 2020;38(15_suppl):6515-.

2. Tao Y, Auperin A, Sun X, Sire C, Martin L, Coutte A, et al. Avelumab-cetuximab-radiotherapy versus standards of care in locally advanced squamous-cell carcinoma of the head and neck: The safety phase of a randomised phase III trial GORTEC 2017-01 (REACH). Eur J Cancer. 2020;141:21-9.

3. Forster MD, Sacco JJ, Kong AH, Wheeler G, Forsyth S, Bhat R, et al. EACH: A randomised phase II study evaluating the safety and anti-tumour activity of the combination of avelumab and cetuximab relative to avelumab monotherapy in recurrent/metastatic head and neck squamous cell cancer. Journal of Clinical Oncology. 2019;37(15_suppl):TPS6091-TPS.

4. Bauman J, Ferris RL, Clump DA, Ohr J, Gooding W, Kim S, et al. LBA36 - Phase I trial of cetuximab, intensity modulated radiotherapy (IMRT), and ipilimumab in previously untreated, locally advanced head and neck squamous cell carcinoma (PULA HNSCC). Annals of Oncology. 2016;27:vi571.

5. Sacco AG, Chen R, Ghosh D, Wong DJL, Worden FP, Adkins D, et al. An open label, nonrandomized, multi-arm, phase II trial evaluating pembrolizumab combined with cetuximab in patients with recurrent/metastatic (R/M) head and neck squamous cell carcinoma (HNSCC): Results of cohort 1 interim analysis. Journal of Clinical Oncology. 2019;37(15_suppl):6033-.

6. Ferris RL, Saba NF, Gitlitz BJ, Haddad R, Sukari A, Neupane P, et al. Effect of Adding Motolimod to Standard Combination Chemotherapy and Cetuximab Treatment of Patients With Squamous Cell Carcinoma of the Head and Neck: The Active8 Randomized Clinical Trial. JAMA Oncol. 2018;4(11):1583-8.

7. Langleben A, Supko JG, Hotte SJ, Batist G, Hirte HW, Rogoff H, et al. A dose-escalation phase I study of a first-in-class cancer stemness inhibitor in patients with advanced malignancies. Journal of Clinical Oncology. 2013;31(15_suppl):2542-.

8. Bendell JC, Hong DS, Burris HA, 3rd, Naing A, Jones SF, Falchook G, et al. Phase 1, open-label, dose-escalation, and pharmacokinetic study of STAT3 inhibitor OPB-31121 in subjects with advanced solid tumors. Cancer Chemother Pharmacol. 2014;74(1):125-30.

9. Wong AL, Soo RA, Tan DS, Lee SC, Lim JS, Marban PC, et al. Phase I and biomarker study of OPB-51602, a novel signal transducer and activator of transcription (STAT) 3 inhibitor, in patients with refractory solid malignancies. Ann Oncol. 2015;26(5):998-1005.

10. Sen M, Thomas SM, Kim S, Yeh JI, Ferris RL, Johnson JT, et al. First-in-human trial of a STAT3 decoy oligonucleotide in head and neck tumors: implications for cancer therapy. Cancer discovery. 2012;2(8):694-705.

11. Cohen EEW, Harrington KJ, Hong DS, Mesia R, Brana I, Perez Segura P, et al. A phase Ib/II study (SCORES) of durvalumab (D) plus danvatirsen (DAN; AZD9150) or AZD5069 (CX2i) in advanced solid malignancies and recurrent/metastatic head and neck squamous cell carcinoma (RM-HNSCC): Updated results. Annals of Oncology. 2018;29:viii372.

12. Rodon J, Curigliano G, Delord J-P, Harb W, Azaro A, Han Y, et al. A Phase Ib, open-label, dose-finding study of alpelisib in combination with paclitaxel in patients with advanced solid tumors. Oncotarget. 2018;9(60).

13. Piha-Paul SA, Knost JA, Braiteh FS, Beck JT, Spitz DL, Papish SW, et al. Genomic mutation profiling (GMP) and clinical outcome of patients treated with buparlisib (PI3K inhibitor) in the “Signature” program. Journal of Clinical Oncology. 2015;33(15_suppl):2516-.

14. Fayette J, Digue L, Ségura-Ferlay C, Treilleux I, Wang Q, Lefebvre G, et al. 1120PD - Buparlisib (BKM120) in refractory head and neck squamous cell carcinoma harbouring or not a PI3KCA mutation: A phase II multicenter trial. Annals of Oncology. 2019;30:v455.

15. Brisson RJ, Dekker A, Souza JAD, Saloura V, Vokes EE, Seiwert TY. A pilot study of the pan-class I PI3K inhibitor buparlisib in combination with cetuximab in patients with recurrent/metastatic head and neck cancer. Journal of Clinical Oncology. 2017;35(15_suppl):6039-.

16. Soulieres D, Faivre SJ, Mesia R, Remenar E, Li S-H, Karpenko A, et al. BERIL-1: A phase II, placebo-controlled study of buparlisib (BKM120) plus paclitaxel in patients with platinum-pretreated recurrent/metastatic head and neck squamous cell carcinoma (HNSCC). Journal of Clinical Oncology. 2016;34(15_suppl):6008-.

17. Bowles DW, Senzer N, Hausman D, Peterson S, Vo A, Walker L, et al. A multicenter phase 1 study of PX-866 and cetuximab in patients with metastatic colorectal carcinoma or recurrent/metastatic squamous cell carcinoma of the head and neck. Investigational New Drugs. 2014;32(6):1197-203.

18. Jimeno A, Bauman JE, Weissman C, Adkins D, Schnadig I, Beauregard P, et al. A randomized, phase 2 trial of docetaxel with or without PX-866, an irreversible oral phosphatidylinositol 3-kinase inhibitor, in patients with relapsed or metastatic head and neck squamous cell cancer. Oral Oncology. 2015;51(4):383-8.

19. Ng M, Tan DS, Subbiah V, Weekes CD, Teneggi V, Diermayr V, et al. First-in-human phase 1 study of ETC-159 an oral PORCN inhbitor in patients with advanced solid tumours. Journal of Clinical Oncology. 2017;35(15_suppl):2584-.

20. Smith DC, Rosen LS, Chugh R, Goldman JW, Xu L, Kapoun A, et al. First-in-human evaluation of the human monoclonal antibody vantictumab (OMP-18R5; anti-Frizzled) targeting the WNT pathway in a phase I study for patients with advanced solid tumors. Journal of Clinical Oncology. 2013;31(15_suppl):2540-.

21. Jimeno A, Gordon M, Chugh R, Messersmith W, Mendelson D, Dupont J, et al. A First-in-Human Phase I Study of the Anticancer Stem Cell Agent Ipafricept (OMP-54F28), a Decoy Receptor for Wnt Ligands, in Patients with Advanced Solid Tumors. Clinical Cancer Research. 2017;23(24):7490.

22. Richter S, Bedard PL, Chen EX, Clarke BA, Tran B, Hotte SJ, et al. A phase I study of the oral gamma secretase inhibitor R04929097 in combination with gemcitabine in patients with advanced solid tumors (PHL-078/CTEP 8575). Investigational New Drugs. 2014;32(2):243-9.

23. Diaz-Padilla I, Hirte H, Oza AM, Clarke BA, Cohen B, Reedjik M, et al. A phase Ib combination study of RO4929097, a gamma-secretase inhibitor, and temsirolimus in patients with advanced solid tumors. Investigational New Drugs. 2013;31(5):1182-91.

24. Massard C, Azaro A, Soria JC, Lassen U, Le Tourneau C, Sarker D, et al. First-in-human study of LY3039478, an oral Notch signaling inhibitor in advanced or metastatic cancer. Annals of Oncology. 2018;29(9):1911-7.

25. Messerschmidt C, Obermayer B, Klinghammer K, Ochsenreither S, Treue D, Stenzinger A, et al. Distinct immune evasion in APOBEC-enriched, HPV-negative HNSCC. Int J Cancer. 2020;147(8):2293-302.

26. El-Khoueiry AB, Desai J, Iyer SP, Gadgeel SM, Ramalingam SS, Horn L, et al. A phase I study of AL101, a pan-NOTCH inhibitor, in patients (pts) with locally advanced or metastatic solid tumors. Journal of Clinical Oncology. 2018;36(15_suppl):2515-.

27. Aung KL, El-Khoueiry AB, Gelmon K, Tran B, Bajaj G, He B, et al. A multi-arm phase I dose escalating study of an oral NOTCH inhibitor BMS-986115 in patients with advanced solid tumours. Investigational New Drugs. 2018;36(6):1026-36.

28. Falchook GS, Dowlati A, Naing A, Gribbin MJ, Jenkins DW, Chang LL, et al. Phase I study of MEDI0639 in patients with advanced solid tumors. Journal of Clinical Oncology. 2015;33(15_suppl):3024-.

29. Chiorean EG, LoRusso P, Strother RM, Diamond JR, Younger A, Messersmith WA, et al. A Phase I First-in-Human Study of Enoticumab (REGN421), a Fully Human Delta-like Ligand 4 (Dll4) Monoclonal Antibody in Patients with Advanced Solid Tumors. Clinical Cancer Research. 2015;21(12):2695.

30. Johnson M, Rasco D, Schneider B, Shu C, Jotte R, Parmer H, et al. Abstract A081: A phase 1b, open-label, dose escalation and expansion study of demcizumab plus pembrolizumab in patients with locally advanced or metastatic solid tumors. Molecular Cancer Therapeutics. 2018;17(1 Supplement):A081.

31. Ferrarotto R, Eckhardt G, Patnaik A, LoRusso P, Faoro L, Heymach JV, et al. A phase I dose-escalation and dose-expansion study of brontictuzumab in subjects with selected solid tumors. Annals of Oncology. 2018;29(7):1561-8.

32. Smith DC, Chugh R, Patnaik A, Papadopoulos KP, Wang M, Kapoun AM, et al. A phase 1 dose escalation and expansion study of Tarextumab (OMP-59R5) in patients with solid tumors. Investigational New Drugs. 2019;37(4):722-30.

33. Rosen LS, Wesolowski R, Baffa R, Liao K-H, Hua SY, Gibson BL, et al. A phase I, dose-escalation study of PF-06650808, an anti-Notch3 antibody–drug conjugate, in patients with breast cancer and other advanced solid tumors. Investigational New Drugs. 2020;38(1):120-30.

34. Jimeno A, Moore KN, Gordon M, Chugh R, Diamond JR, Aljumaily R, et al. A first-in-human phase 1a study of the bispecific anti-DLL4/anti-VEGF antibody navicixizumab (OMP-305B83) in patients with previously treated solid tumors. Investigational New Drugs. 2019;37(3):461-72.
